# Supplementary material for: Content-rich biological network constructed by mining PubMed abstracts
Source: BMC Bioinformatics. 2004 Oct 8;5:147. doi: 10.1186/1471-2105-5-147 (PMC528731; doi:10.1186/1471-2105-5-147)
Supplement: Additional File 2 — The original results of the above study (non-essential files are deleted to keep the file size under the limit set by BMC bioinformatics). [file 1471-2105-5-147-S2.bz2 › chilibotAdditionalFile2/dip10/317ID10797014E2428/html/UL18_UL38.html]

 


 **UL18** and **UL38** 
  
Found 8 abstracts in PubMed, retrieved 8.  
 

 What does Google say? 
 PDF only 
| .edu only 

---

**Interactive relationship** (e.g. stimulation, inhibition, etc)

**Non-interactive relationship** (e.g. studied together, co-existance, homology, etc.)

- These and other observations indicate that i the products of the  **UL18** , UL19, UL35, and  **UL38**  genes self assemble into structures that form the outer surface icosahedral shell of the capsid, ii the products of the UL26 and or UL26.5 genes are required as scaffolds for assembly of 100 nm capsids, and iii the interaction of the outer surface of the capsid with the scaffolding proteins requires the product of the  **UL18**  gene VP23.  Ref: 8139029 J Virol, 1994
- coded by the UL19 gene and two minor proteins, VP19C  **UL38**  gene and VP23  **UL18**  gene.  Ref: 8918599 J Mol Biol, 1996
- In infections in which single capsid genes were left out, it was found that the  **UL18**  VP23, UL19 VP5,  **UL38**  VP19C, and either the UL26 VP21 and VP24 or the UL26.5 VP22a genes were required for assembly of 100 nm capsids.  Ref: 8139029 J Virol, 1994
- The product of  **UL38**  has been shown to be essential for capsid assembly, but no role has previously been assigned to the product of  **UL18** .  Ref: 2161052 J Gen Virol, 1990
- The sequences of two capsid proteins VP19C and VP23 showed them to be encoded by genes  **UL38**  and  **UL18** , respectively.  Ref: 2161052 J Gen Virol, 1990
- Recombinant baculoviruses were used to express the six capsid genes  **UL18** , UL19, UL26, UL26.5, UL35, and  **UL38**  in insect cells.  Ref: 8139029 J Virol, 1994
- Herpes simplex virus type 1 HSV 1 intermediate capsids are composed of seven proteins, VP5, VP19C, VP21, VP22a, VP23, VP24, and VP26, and the genes that encode these proteins, UL19,  **UL38** , UL26, UL26.5,  **UL18** , UL26, and UL35, respectively.  Ref: 7745718 J Virol, 1995
- VP5, VP19C, VP22a and VP23 have been shown previously to be the products of genes UL19,  **UL38** , UL26.5 and  **UL18** , respectively.  Ref: 1328483 J Gen Virol, 1992
- The proteins coded by the five major capsid genes of herpes simplex virus 1, VP5 gene UL19, VP19c  **UL38** , VP23  **UL18** , pre VP22a UL26.5, and pre VP21 UL26, assemble into fragile roundish procapsids, which mature into robust polyhedral capsids in a transition similar to that undergone by bacteriophage proheads.  Ref: 8918600 J Mol Biol, 1996
- The proteins encoded by  **UL18**  VP23, UL19 VP5, UL35 VP26 and  **UL38**  VP19C are components of the outer capsid shell whereas those specified by UL26 VP21 and VP24 and UL26.5 VP22a, are involved in scaffold formation.  Ref: 8176371 J Gen Virol, 1994
